# The Use of 3D Printing Technology for Manufacturing Metal Antennas in the 5G/IoT Context

**DOI:** 10.3390/s21103321

**Published:** 2021-05-11

**Authors:** Diogo Helena, Amélia Ramos, Tiago Varum, João N. Matos

**Affiliations:** 1 Instituto de Telecomunicações, Campus Universitário de Santiago, 3810-193 Aveiro, Portugal; diogo.helena@ua.pt (D.H.); ameliaramos@ua.pt (A.R.); matos@ua.pt (J.N.M.); 2 Department of Electronics Telecommunications and Informatics, University of Aveiro, Campus Universitário de Santiago, 3810-193 Aveiro, Portugal

**Keywords:** 3D printing, metal antennas, horn antennas, IoT sensors, 5G

## Abstract

With the rise of 5G, Internet of Things (IoT), and networks operating in the mmWave frequencies, a huge growth of connected sensors will be a reality, and high gain antennas will be desired to compensate for the propagation issues, and with low cost, characteristics inherent to metallic radiating structures. 3D printing technology is a possible solution in this way, as it can print an object with high precision at a reduced cost. This paper presents different methods to fabricate typical metal antennas using 3D printing technology. These techniques were applied as an example to pyramidal horn antennas designed for a central frequency of 28 GHz. Two techniques were used to metallize a structure that was printed with polylactic acid (PLA), one with copper tape and other with a conductive spray-paint. A third method consists of printing an antenna completely using a conductive filament. All prototypes combine good results with low production cost. The antenna printed with the conductive filament achieved a better gain than the other structures and showed a larger bandwidth. The analysis recognizes the vast potential of these 3D-printed structures for IoT applications, as an alternative to producing conventional commercial antennas.

## 1. Introduction

The fifth generation of mobile communications (5G) is driven by an unprecedented growth in the number of connected devices and shared data [1]. With the main goal of being a unifying connectivity structure for the next decade and beyond [2], 5G enables the IoT reality, where a device will be able to maintain connectivity, regardless of time or location. Everything will be monitored, measured, or sensed, and to gather that information, the number of devices interacting with the surroundings will increase exponentially.

New scenarios such as the proliferation of sensors to deliver IoT services associated to home appliances, health monitoring, smart offices, efficient navigation systems (autonomous cars), immersive multimedia experiences, either through augmented or virtual reality and cloud computing, will all be combined in a typical 5G network [1]. Given the number of the connected devices, their diversity of nature, sizes, and shapes, the antennas will face multiple challenges with various forms and the combination of several materials.

New techniques to build antennas need to be investigated, to properly create radiation structures in the daily objects. Technology must be able to handle heterogeneous and challenging layouts, always bearing in mind the improvement of both energy and cost efficiencies along with spectrum performance [3].

Wider bandwidths are probably the most effective method to provide the data demands for 5G services [4], thus the migration to the millimeter waves region becomes mandatory. Several concerns arise with these operation frequencies, essentially due to the huge path-loss and consequent fragile link result of the occurring diffractions [1]. To overcome these communication issues, combating the large propagation loss in mmWaves, it is important to use high gain and highly directional antennas. Besides, it is highly recommended that antennas are compact, robust, and with a low-cost and fast production.

When compared with dielectric antennas, metal antennas, such as horn, dipoles, loop, slots, etc., due to the low losses of metals, typically allow to obtain more efficient radiating structures, greater gains (as singular elements), and even enable handling greater power levels. However, their costs, manufacturing process, and weights are limiting factors to their extensive use.

Within this context, horn antennas are radiating structures with relatively high gain and wide bandwidth, while being very robust [5]. These structures are widely used as a feed element for large radio astronomy, satellite tracking, and communication dishes, and they can be found installed throughout the world [6]. Their characteristics, associated with the simplicity of construction, make them suitable for application in sensors or even integration in larger antenna arrays.

Empowered by many demanding commercial and aerospace markets, recent advances in 3D printing technology have been tested as an alternative to the more traditional processes, such as milling or injection molding. Both techniques have advantages, but also major disadvantages when compared to 3D printing. The milling process results in a large amount of wasted metal scrap, and on the other hand, molding injection is very time-consuming and has higher production costs [7].

3D printing presents itself as a more environment-friendly alternative, a crucial aspect in the 5G’s context, given the goal to become the greener mobile communications generation ever. Moreover, this manufacturing process has the advantages of minimizing the time-to-market (allowing for fast prototyping), not causing waste, and presenting endless versatility of the structures created, making possible to create highly complex shapes [8].

The combination of the potentiality of metal-radiating structures, with the benefits of 3D printing technology, is a promising solution in the context of 5G communication systems, and their main challenges. In [9] a stepped reflector was built from a laser sintering process. This work confirms the viable option of 3D printing technique to build high frequency and medium-sized antennas. Despite presenting proper gain and good efficiency, the construction process makes this strategy prohibitive, because it is very cumbersome and a spray coating is necessary to apply. One of the main limitations of 3D printing is the ability to produce metallic elements [10]. In this sense, a liquid metal alloy was used to produce a Yagi-Uda Loop Antenna, operating at 2.45 GHz using a low-cost manufacturing process. The material used to print the structure was the resin FormLabs Durable.

Regarding horn antennas, three prototypes operating at X band were produced with 3D printing technology in [11]. The antennas were printed in ABS and coated with silver paint, copper tape, and were copper plated. These prototypes were further compared with a conventional aluminum horn antenna with the same dimensions. The antenna that was copper plated had the best gain value. In [12] a wide variety of components were manufactured operating in the Ku-band. Nonetheless, a horn antenna with 17 dBi gain and good radiation patterns was obtained, and authors point out the antennas’ surface roughness as the main reason to justify the losses found in the measured gain. For the Ka-band, in [13], two identical horn antennas were 3D printed. One was painted with a copper conductive paint, and the other one was covered with copper tape. These prototypes presented simulated gain values of 18.6 dBi and 9.4 dBi, for the copper painted and copper tape antennas, respectively, despite 28 GHz measurements were not performed. This significant difference is due to the coating process used. The reference [14] shows good results for a corrugated horn antenna operating in the X band, with a structure manufactured using an ABS material, and coated using copper with electroless metallization method. In [15] two slot antennas are proposed for operating at 28 GHz. These antennas required a post-printing treatment with a spray coating. Authors claim that this metallization method allows a lower cost of fabrication when compared to the conventional metallization processes. Nevertheless, this coating technique presents a significant issue since it is not possible to assure a thickness uniformity across the whole structure.

Also, for the Ka-band, an attractive metallization process was tested, requiring no treatment process before and after 3D printing [16]. The printed structure was metallized using electrodeposition of silver conductive ink and the manufactured prototype achieved a good performance. The drawback of this technique is that the coating process produced a non-uniform layer of paint.

Structures that do not require a post-printing treatment process have received great attention, irrespective of the structures being printed with conductive materials or directly printed on metal [17,18]. In [19], two-horn antennas have been modelled using 3D printing, operating at 12 GHz. One was printed with a regular filament and covered with copper tape while the other was directly printed with ProtoPasta, a conductive PLA. The first prototype obtained very promising results, both in bandwidth and measured gain. On the other hand, the prototype printed with the conductive material obtained a low gain when compared with the typical values of horn antennas and an efficiency of only 35%. Therefore, ProtoPasta does not prove to be the most suitable material for applications within the context of the new mobile communication systems.

To date, there are a few studies on 3D printing with conductive filaments [20,21,22,23]. However, in all these works the operating frequency is low when compared to the 5G demands. Regarding the conductive filaments, an effort was made in the parametric study of 3D printing specifications for use in printing topologies with the Electrifi material [24]. Although a microstrip patch antenna and a pyramidal horn antenna were correctly built with Electrifi [24], respectively for the operating frequencies of 2.5 GHz and 5.8 GHz, there are some challenges in the production of RF devices for higher frequencies using this type of material. In [25,26,27] three different antennas were developed using Electrifi filament with conductive properties, an electric meandered dipole antenna, a 3D-printed dipole, and a 3D-printed conformal patch antenna, for 915 MHz, 900 MHz, and 2.32 GHz respectively.

A prototype of a 3D metal printed polarization reconfigurable horn antenna is presented in [28] for K/Ka band, using titanium material, revealing a reasonable agreement between simulations and measurements. A low-profile all-metal antenna design with resonant cavity is described in [29] to operate in the frequency range of 130–150 GHz. To create the conductive layer, the antennas were coated with a thin layer of platinum using a physical vapor deposition. Another prototype was made using direct metal laser sintering. Both processes present a huge complexity and cost.

This paper presents and compares a set of techniques for the development of typically metallic antennas using 3D printing. In this sense, as an example, several horn antennas are developed to operate at 28 GHz, for 5G/IoT communications, using different methods, these methods can be replicated to other different structures.

The article is divided into six sections, starting with an introduction with a state-of-the-art report, where possible applications for the prototypes proposed are presented and the objectives of this work are settled. In the Section 2, all construction methods are described, along with the materials used in each prototype. In Section 3 the simulation and measured results of all designed antennas are shown. Next, the Section 4 presents an analysis of the results obtained. Finally, in Section 6, the main conclusions are reported.

## 2. Basis Antenna Structure

In this work, the analysis and description of the various techniques for the construction of typically metallic radiating elements using the potential of 3D printing will be supported by the construction of a typical structure which offers reasonable high bandwidths and gain values, which are the horn antennas. However, it can be extended to other types of metal structures.

**Figure 1:**
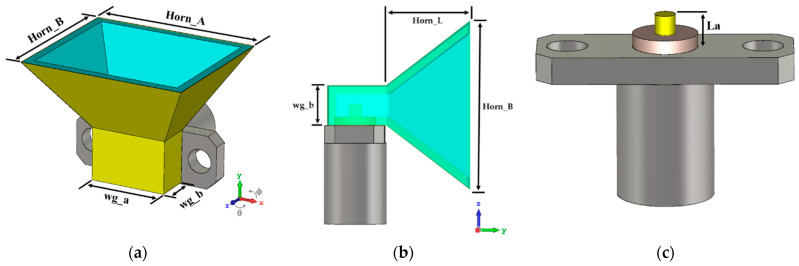
Geometry of the horn antenna: (**a**) three-dimensional view, (**b**) profile view, and (**c**) SMA connector operating as a monopole for the feeding structure.

Horn antennas are one of the simplest antennas in the microwave frequency bands. Traditionally, these antennas are made of metallic materials and are typically fed by a section of a waveguide. Their structure forms a smooth transition between the waveguide and the free space, directing the radio waves into a beam [30]. Horn antennas may adopt several forms and the most common types are the rectangular, or pyramidal, and the conical. Rectangular horns start from a rectangular waveguide propagating the TE10 mode, and in these cases, one face is enlarged with a pyramidal shape, as can be seen in Figure 1a,b. Due to its ease of construction, wide bandwidth, versatility, ease of excitation, and high gain, this type of antenna is used in multiple applications.

In this work, the pyramidal structure was selected, mainly due to the associated simplicity of construction. To properly design the antenna, some aspects were considered, regarding gain, operating frequency, and the feeding waveguide dimensions (wg_a × wg_b).

The waveguide’s dimensions were found based on the standard rectangular waveguide WR34 (8.636 mm × 4.318 mm, which operates from 22 GHz up to 33 GHz). Regarding the size of the horn, it was calculated to operate at 28 GHz and perform a gain of G = 12 dBi. In horn antennas the gain is the starting point to calculate its aperture dimensions. Bearing in mind that it is important to obtain a compact prototype, this gain was chosen as a tradeoff for a value that would not represent a prototype too large. Considering the design parameters shown in Figure 1, the theoretical models [31] were used to estimate the initial dimensions of the horn antenna.

The preliminary values of the horn were submitted to an optimization process using the CST simulator. Although the waveguide was based on a standard, the versatility of the 3D printing allows to optimize its dimensions for this specific application.

**Figure 2:**
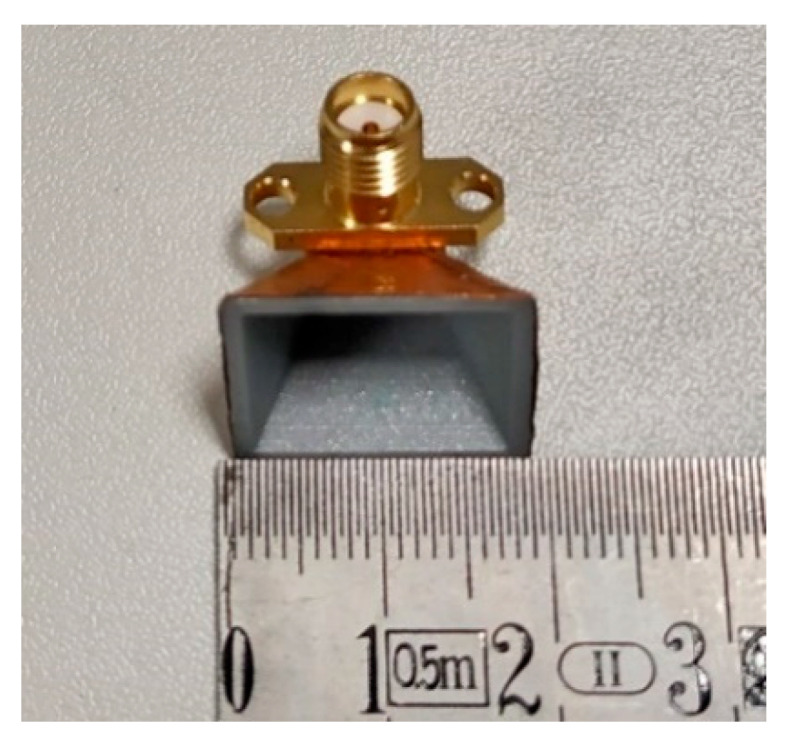
3D-printed horn antenna with copper tape.

Horn antennas are typically fed by a radiation element that is placed at a *λ/4* distance from the waveguide’s closed face. There are a variety of possibilities to excite a horn antenna, however a slot or a monopole element are the most common methods. In this work, and for simplicity, RS- Pro-27GHz SMA connector (two hole flange mount) operating as a monopole antenna was used, as it is shown in Figure 2. Its inner pin has a length of *La* and acts as a monopole at 28 GHz, properly feeding the structure.

## 3. Proposed Metallization Techniques

To analyze the different techniques of producing an antenna using the 3D printing technology, three approaches were adopted, using copper tape, conductive paint, and conductive filament. All antennas were printed with an Ultimaker 3 Extended printer [32], which is based on the fused deposition modelling (FDM) technique. Their design process and construction methods are presented in the following sections.

### 3.1. Horn Antenna with Copper Tape Metallization

The process started by printing the optimized horn antenna structure of Figure 1 with regular PLA, which is the most common material within the 3D printing filaments. After printing, the antenna was covered with a single layer of copper tape.

The PLA used in this process was from the Ultimaker supplier and it possesses a dielectric constant *ε_r_* of 2.7 and a dissipation factor *tg(δ)* of 0.008 @ 1 MHz [19]. The copper tape was from Würth Elektronik and has a thickness of 0.04 mm. It is important to highlight that in the case of this particular tape, its glue has electrical conductivity properties. The final parameters of the antenna’s dimensions are presented in Table 1 and the produced prototype is shown in Figure 2. The wall thickness of the antenna is 0.8 mm.

### 3.2. Horn Antennas with Copper Conductive Paint

The construction process of these antennas is similar to that previously described, except that instead of covering the structure with copper tape, a conductive spray-paint is applied on the top of the PLA structure.

The commercial spray used for coating these antennas was the RS EMI/RFI Shielding Aerosol 400 mL [33]. This is a spray that is easy to apply and with a drying time of approximately 5 min. However, it reaches its maximum conductivity after 24 h of its application.

Several prototypes were assembled, with small modifications, regarding the number of conductive layers, and the roughness of the structure walls, to analyze their impact in the performance of the antenna and therefore, to study the best technique to fabricate this type of antenna. Five prototypes were printed, where in three of them the spray was directly applied to the antenna without any preparation. The other two prototypes were previously sanded to achieve a smoother surface, diminishing the roughness among printed layers, where the paint would be applied.

With the first three models it was intended to analyze the impact of the variation of the number of layers of conductive spray-paint applied, and thus, two, three, and four layers of the above-mentioned spray coating were applied. In the sanded prototypes two and three layers of the same spray-paint were applied.

**Figure 3:**
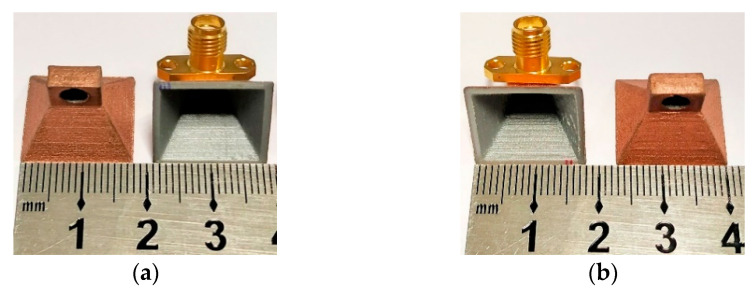
3D-printed horn antennas with conductive paint: (**a**) without sanding process and (**b**) with sanding process.

Figure 3 shows two of the prototypes made with conductive paint using each technique, without the sanding process, Figure 3a, and with the sanding process, Figure 3b.

### 3.3. Horn Antennas Built from Conductive Filament

Typically, antennas produced with 3D printing technology require treatment after printing, such as the metallization of their structure. Given the increasing advancement in technology and the widespread use of mobile communications, it becomes necessary to develop antennas that are easy and fast to produce in mass. Therefore, a possible solution would be to produce an antenna entirely with a conductive filament.

The material used to produce the designed horn structure exclusively with the 3D printing process was the Electrifi, a conductive filament from Multi3D [34]. As mentioned in [34], this material is considered as the most conductive filament available on the market and has an electrical conductivity of σ = 1.67 × 10^4^ S/m, making its usage highly promising for the design of radiating structures for mmWaves.

**Figure 4:**
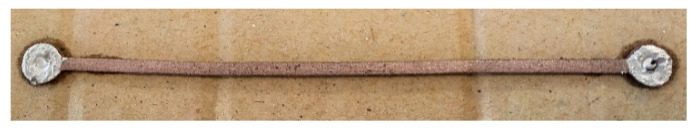
Printed line with Electrifi material.

To confirm the proper conductivity of Electrifi, it was characterized. A line with dimensions 0.2 cm × 10 cm × 0.2 cm was printed, as shown in Figure 4. Epoxy EPO-TEK H20E from Epoxy technology [35] was used to ensure the electrical contact between the multimeter terminals and the printed part.

Several measurements of the material DC resistance were performed, and the values obtained are shown in Table 2. Using (1) and (2), where *R* is the resistance of the material, *ρ* is the resistivity, σ is the conductivity, *l* represents the line length, and *A* is the respective section, it was possible to obtain an estimation of the conductivity value of σ = 2.22 × 10^3^ S/m. The difference between this value and the one mentioned by the manufacturer may be because this resistance is of a printed piece and not of the material itself, as mentioned in [34]. Table 3 holds all the estimated properties of the referred material, which were considered on the antenna design process.

(1) $$R=\rho \frac{l}{A}\;\left(\Omega /m\right)$$

(2) $$\sigma =\frac{1}{\rho }\;\left(S/m\right)$$

Despite the Electrifi supplier suggests the most suitable printing settings for this material, for high frequencies, the printing process can be a challenging task since the structure to be printed has a small size. After some manufacturing tests, the printing parameters have been set, and are presented in Table 4.

It should be noted that this prototype was printed in vase mode. This mode consists of a constant printing, in which the nozzle goes through layer by layer in a spiral form as if forming a vase. This solution not only allowed the use of less material but significantly optimized the printing time, with the prototype being printed in approximately 12 min.

**Figure 5:**
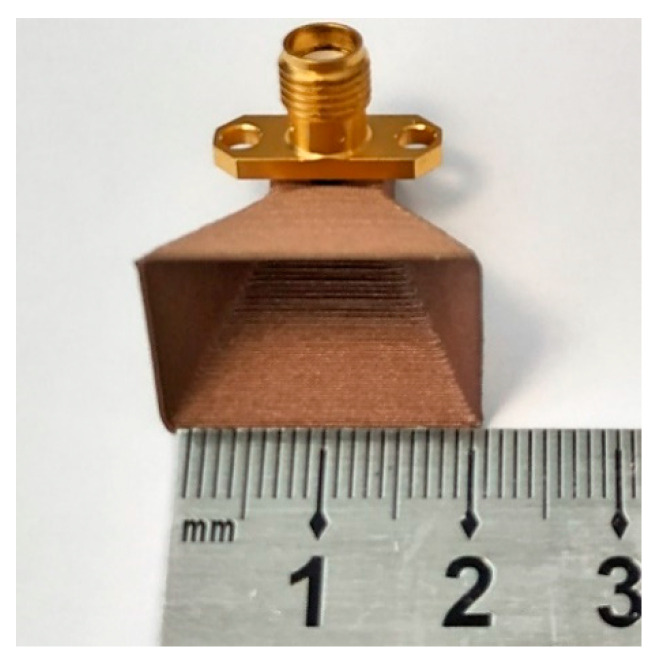
3D horn antenna printed with conductive filament.

The prototype printed with Electrifi has a wall thickness of 0.4 mm. The final dimensions, listed in Table 5 and Figure 5, show the antenna built using the conductive filament.

## 4. Results

The constructed prototypes using the different techniques to metallize the surface of the antenna were tested and measured, and their results were compared with the ones obtained from the simulated models. Measurements were performed with a VNA that operates up to 67 GHz (E8361C by Agilent Technologies, Santa Clara, CA, USA) and two reference antennas (LB-180400-KF 18–40 GHz Broadband Horn Antenna), for radiation pattern and gain, ensuring the antennas in the far-field.

### 4.1. Horn Antenna with Copper Tape Metallization

**Figure 6:**
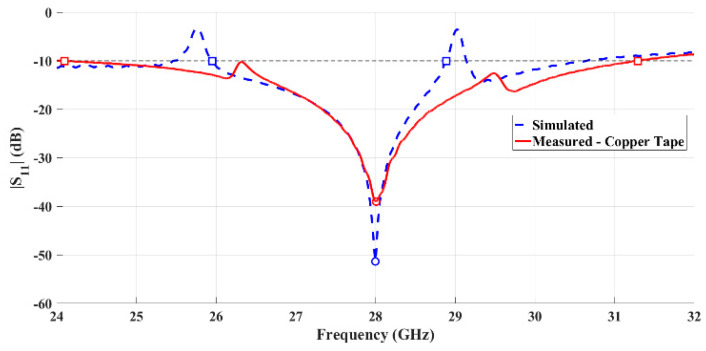
Comparison between simulated and measured reflection coefficient of the fabricated horn antenna using copper tape.

Figure 6 shows the comparison between the simulated and measured reflection coefficient of the copper-taped horn antenna. It is possible to observe that the measured and simulated results are clearly in agreement, and the antenna is properly matched, with the minimum value of S_11_ appearing at 28 GHz.

Considering the S_11_ < −10 dB criteria, the antenna exhibits a measured bandwidth of 7.19 GHz (26.0%). Comparing this result with those simulated, 2.93 GHz (10.7%), an improvement is observed.

**Figure 7:**
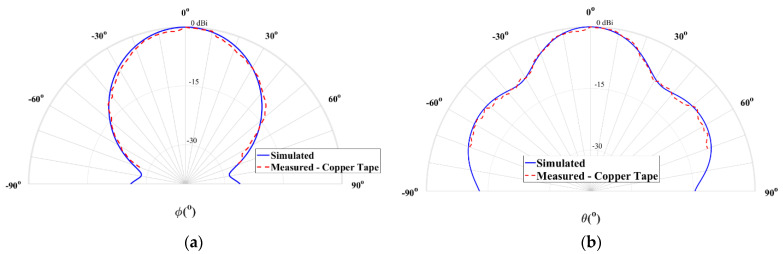
Simulated and measured normalized radiation pattern at 28 GHz of the horn antenna with copper tape: (**a**) plane θ = 90° and (**b**) plane ϕ = 90°.

Figure 7 shows the comparison between the simulated and measured radiation patterns of the horn antenna using copper tape, for the two main radiation planes, θ = 90° and ϕ = 90°.

It is possible to observe that the measurement results are close to the simulations. This antenna exhibits a measured half power beamwidth (HPBW) of 38° in the plane θ = 90°, while in the orthogonal plane (ϕ = 90°) is about 33°. The copper-taped horn antenna has a simulated gain of 12 dBi and a measured gain of 11.2 dBi, at 28 GHz, which is a clearly satisfying result.

### 4.2. Horn Antennas with Copper Paint

Using this method, two approaches were tested with effect mainly on the finishes, which are with or without sanding of the 3D structure surface.

**Figure 8:**
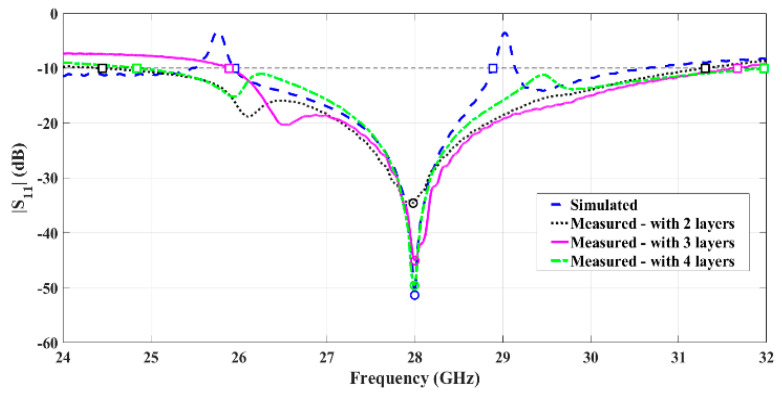
Comparison between simulated and measured reflection coefficient of the fabricated horn antennas using conductive paint, with different numbers of ink layers.

• Without sanding process:

Three prototypes were built using a different number of layers of conductive ink and characterized by their main parameters. Figure 8 represents the comparison of the simulated and measured reflection coefficient of the painted antennas without the sanding process.

Analyzing these results, it can be observed that all prototypes without the sanding process are well matched, and the minimum value of S_11_ happens at 28 GHz. It is also possible to observe that by increasing the number of layers, the antenna adopts a behavior closer to the simulations, improving even more its matching. Moreover, the prototype with four layers presents roughly 7.13 GHz of bandwidth, from 24.84 GHz up to 31.97 GHz, making this a very promising result.

**Figure 9:**
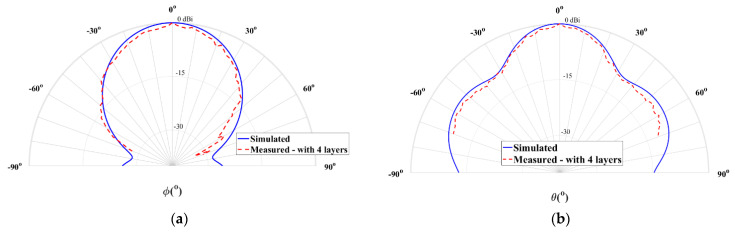
Simulated and measured normalized radiation pattern at 28 GHz of the 4-layer horn antenna with conductive paint: (**a**) plane θ = 90° and (**b**) plane ϕ = 90°.

Figure 9 shows the comparison of the simulated and the measured normalized radiation pattern of the antenna coated with four layers of spray-paint without sanding.

According to Figure 9, a good agreement between both curves is noticeable. The antenna has a HPBW of 34.2° and 29° in the planes θ = 90° and ϕ = 90°, respectively, with a measured gain of 10.8 dBi at 28 GHz, and reminding that the simulated value was 12 dBi, which is a satisfactory result.

**Figure 10:**
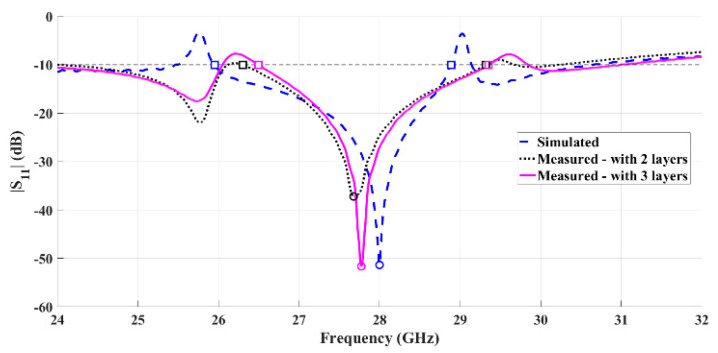
Comparison between simulated and measured reflection coefficient of the fabricated horn antennas using conductive paint, after applying sanding process.

• With sanding process:

To evaluate the roughness impact of this manufacturing process on the antenna’s characteristics, the two painted prototypes were compared applying the sanding process in the structure. Figure 10 presents the comparison between the simulated and measured reflection coefficient of the two and three-layer painted antenna after a hand-made sanding process, which is not accurate.

Although both prototypes that were submitted to the sanding process exhibit similar behavior to the simulated antenna, there is a slight deviation from the resonant frequency. This may be due to the sanding process, since small variations in the antenna’s structure influence its performance. To compensate this effect, it is suggested to superficially increase the external antenna’s dimensions.

**Figure 11:**
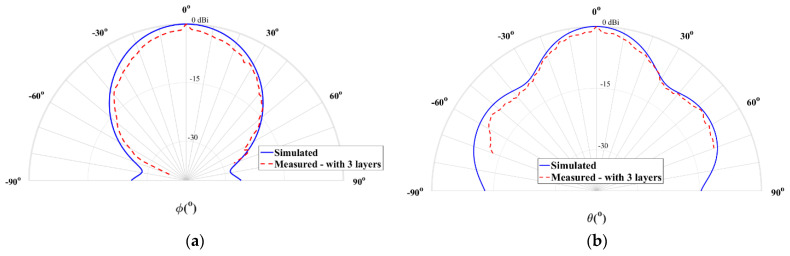
Simulated and measured normalized radiation pattern at 28 GHz of the three-layer horn antenna (sanded) with conductive paint: (**a**) plane θ = 90° and (**b**) plane ϕ = 90°.

The sanded antenna covered with three layers has a bandwidth of 2.86 GHz (10.2%) clearly close to the 2.93 GHz of simulations. The measurements of the two main radiation planes of this prototype were performed and are shown in Figure 11.

It can be observed, and in agreement with the other antennas, both radiation patterns are similar. Based on the high directivity observed and the gain of 11.8 dBi, these are satisfactory results for a 3D-printed horn antenna. The measured HPBW was 26.7° and 27° in the planes θ = 90° and ϕ = 90°, respectively.

### 4.3. Horn Antennas with Conductive Filament

**Figure 12:**
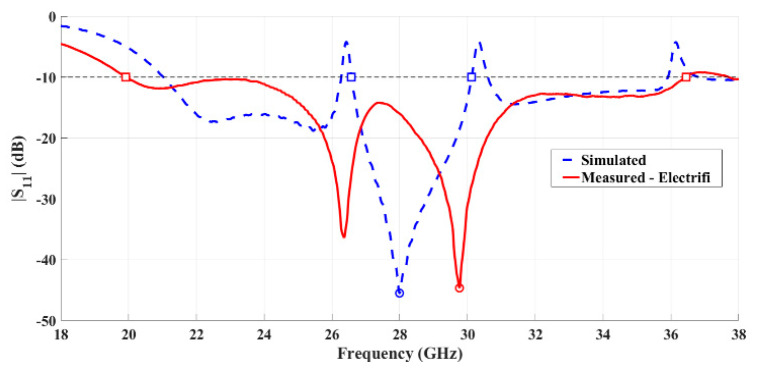
Comparison between simulated and measured reflection coefficient of the fabricated horn antenna using conductive filament.

Regarding the 3D-printed horn antenna using the conductive material, the comparison between the simulated and measured reflection coefficient is shown in Figure 12.

Although the minimum value of S_11_ occurs at 29.76 GHz, a reasonable level of impedance matching was obtained since for the desired operating frequency, the practical S_11_ is 16.02 dB. Regarding the obtained bandwidth, a significant improvement was verified. While in simulation a bandwidth of 3.55 GHz was found, the measured bandwidth of 16.52 GHz (58.6%) was accomplished.

**Figure 13:**
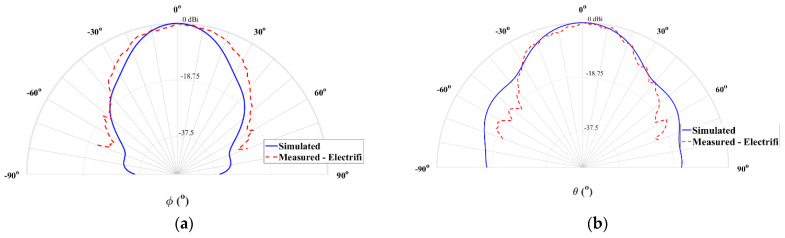
Simulated and measured normalized radiation pattern at 28 GHz of the 3D-printed horn antenna with conductive filament: (**a**) plane θ = 90° and (**b**) plane ϕ = 90°.

Lastly, the normalized radiation pattern of this prototype is presented in Figure 13.

It is possible to notice that the measured radiation pattern presents an agreement compared with the obtained through simulation, mainly in boresight. Furthermore, the 12 dBi of measured gain proves that this material is suitable for this operating frequency, allowing its implementation in antenna structures for emerging communications systems. In the plane θ = 90°, the simulated HPBW was 27° while its measured value was 38.4°. Regarding the plane ϕ = 90°, the simulated HPBW was 32° while 31.5° was measured.

### 4.4. Additional Results

**Figure 14:**
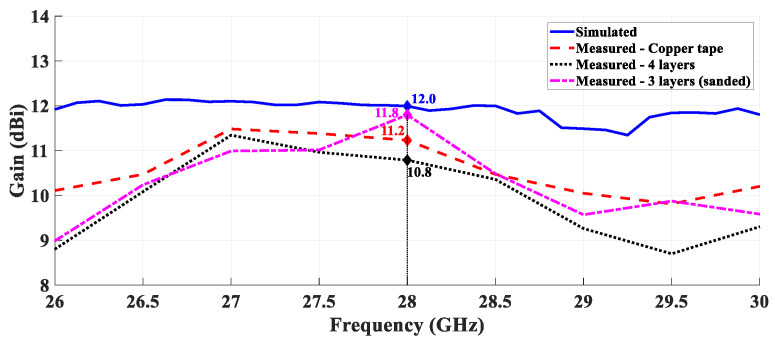
Comparison between simulated and measured gain over the frequency—PLA structure covered with copper.

**Figure 15:**
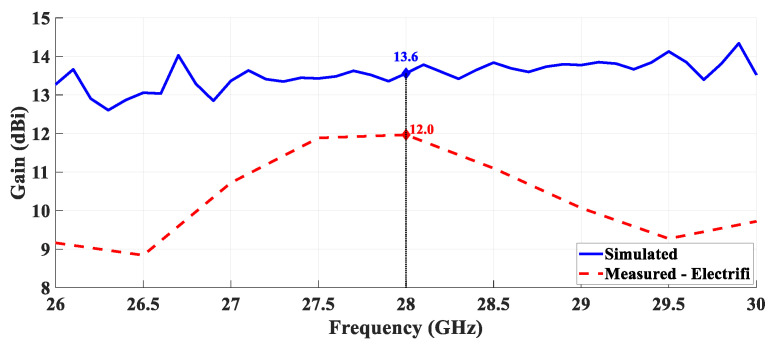
Comparison between simulated and measured gain over the frequency—horn antenna printed with the conductive filament.

A comparison of the simulated and measured gain over the frequency was performed, for the PLA structure covered with copper and for the Electrifi structure. These results are presented in Figure 14 and Figure 15, respectively.

According to Figure 14 it is possible to confirm that the gain as a function of the frequency of the three metallized antennas is similar and agrees with the simulated results. Observing the Figure 15 it is possible to evidence the electrical conductivity of the Electrifi filament and the proper performance for this operating frequency.

**Figure 16:**
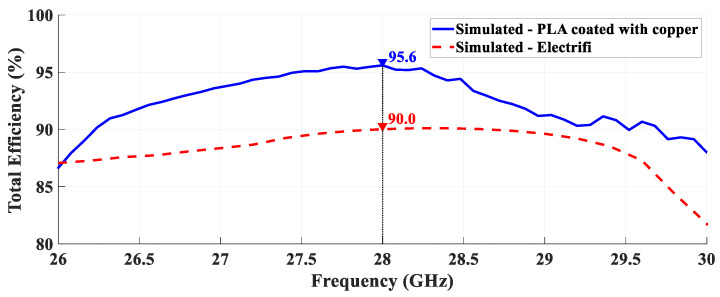
Simulated total efficiency of both antennas.

Figure 16 shows the simulated total efficiency over the frequency of both antennas.

It is possible to observe that the horn antenna printed with PLA and covered with copper has a maximum efficiency of 95.6% at the desired frequency, 28 GHz. On the other hand, the prototype fabricated with the Electrifi material presents 90.0% of total efficiency and although this result was obtained in a simulation environment, it is a promising one, especially given the novelty associated with the conductive filaments.

## 5. Discussion

3D printing is a common technique to all antennas produced, nevertheless, different approaches were followed in this work. In all prototypes, the results obtained were satisfactory and quite promising bearing in mind the requirements of the next generation of mobile communications.

The production cost was estimated considering the percentage of material used in the production of the prototype relative to the total cost of the materials used in the structure of each prototype, without taking into account the cost of the connector, confirming the advantage of 3D printing in the reduction of manufacturing cost when compared with conventional techniques. Table 6 summarizes the main measured results of the developed prototypes, along with their estimated production cost and total weight.

It is possible to verify that the first two prototypes present very similar values, both in bandwidth and in measured gain.

The minor differences between both results, we believe are due to the behavior of both coating layers, their losses, and their interaction with the base PLA structure. While the copper strip has a homogeneous structure on the PLA, being unaffected by the possible imperfections in the PLA, such as pores or small roughness, the PLA structure covered with paint does not guarantee a completely homogeneous structure, without pores or with a uniform layer.

On the other hand, and despite the acceptable gain value, the antenna that was submitted to the sanding process presents a bandwidth lower than all the other prototypes, but it is the closest to the simulation. Finally, the antenna produced with the conductive filament has a considerably higher bandwidth than the other prototypes.

Concerning production costs, the antenna with the highest cost is the last prototype made of conductive filament due to the initial cost of the material used. In the context of the available 3D filaments, and more specially in the electrically conductive ones, Electrifi is truly revolutionary and thus its higher price. Nevertheless, the overall cost of the antenna production remains low.

All prototypes built have reduced weight and costs compared to a metal-based antenna with similar characteristics, weighing at least ten times more and being sold with cost of approximately 1500 euros.

## 6. Conclusions

3D printing is considered to be a technology with greater change for development and integration in emerging communication systems. This technology has several advantages over conventional part-making techniques, with rapid prototyping and low cost being the most notable. In this article, different techniques for creating metal antennas with the aid of 3D printing were presented and applied to the typical case of a horn antenna. A total of seven samples were produced, and their main characteristics were analyzed, combining the low-cost manufacturing, the simplicity of the structure with the positive results achieved. The sanded antenna coated with conductive paint has a lower bandwidth despite having a higher gain, when compared to the prototype using copper tape and that without the sanding process. On the other hand, although the antenna using conductive filament is the one with higher production costs, it has in fact not only a better gain, but also a significantly improved bandwidth. It also has the advantage that no processing is required after printing.

Overall, the results are promising and point to 3D printing technology as a good choice for communication applications to provide a possible cost-effective solution for commercial applications.

## Figures and Tables

**Table 1:** Dimensions of 3D-printed horn antenna coated with copper tape.

Parameters	wg_a	wg_b	Horn_A	Horn_B	Horn_L	La
Dimensions (mm)	8.18	3.85	17.9	16	8	2.05

**Table 2:** Resistance of the Electrifi material.

Measures	R1	R2	R3	R4	R5	R6	$\bar{R}$
Resistance (mm)	11.3 Ω	11.0 Ω	11.0 Ω	11.5 Ω	11.3 Ω	11.2 Ω	11.22 Ω

**Table 3:** Characterization of the Electrifi material.

Length *l* (cm)	Section *A* (cm^2^)	Resistance *R* (Ω)	Resistivity *ρ* (Ω·cm)	Conductivity *σ* (S/m)
10	0.04	11.22	0.045	2.22 × 10^3^

**Table 4:** 3D-printing settings for PLA and Electrifi filaments.

Printing Setting	PLA	Electrifi
Printing speed	70 mm/s	15 mm/s
Printing temperature	200 °C	140 °C
Layer height	0.3 mm	0.2 mm

**Table 5:** Dimensions of 3D-printed horn antenna using conductive filament.

Parameters	wg_a	wg_b	Horn_A	Horn_B	Horn_L	La
Dimensions (mm)	8.35	3.85	22	17.15	13.25	2.15

**Table 6:** Comparison of the different prototypes.

Antenna	Bandwidth	Gain@ 28 GHz	Production Cost	Total Weight
Copper tape	7.19 GHz (26.0%)	11.2 dBi	0.45 €	2.6 g
4 layers of paint	7.13 GHz (25.1%)	10.8 dBi	0.18 €	2.3 g
3 layers of paint (sanded)	2.86 GHz (10.2%)	11.8 dBi	0.14 €	2.3 g
Electrifi	16.52 GHz (58.6%)	12.0 dBi	1.78 €	2.5 g

## Data Availability

Not applicable.
